# Monomeric C-reactive protein affects cell injury and apoptosis through activation of p38 mitogen-activated protein kinase in human coronary artery endothelial cells

**DOI:** 10.17305/bjbms.2020.4711

**Published:** 2020-11

**Authors:** Yong Zhang, Hongxia Cao

**Affiliations:** 1 Department of Vasculocardiology, The First Affiliated Hospital of Anhui Medical University, Hefei, China; 2 Department of Nephrology, The First Affiliated Hospital of Anhui Medical University, Hefei, China

**Keywords:** C-reactive protein, cell injury, cell apoptosis, p38 mitogen-activated protein kinase, human coronary artery endothelial cells

## Abstract

C-reactive protein (CRP) is an important predictor of cardiovascular events and plays a role in vascular inflammation and vessel damage. The aim of this study was to investigate the effect of pentameric CRP (pCRP) and monomeric CRP (mCRP) on the production of atherosclerosis-related factors in cultured human coronary artery endothelial cells (HCAECs). HCAECs were treated with pCRP, mCRP, p38 mitogen-activated protein kinase (MAPK) inhibitor SB203580, or transfected with p38 MAPK siRNA. Western blotting was performed to detect the expression of vascular endothelial growth factor (VEGF), cyclooxygenase-2 (COX-2), intercellular adhesion molecule-2 (ICAM-2) and vascular cell adhesion molecule-1 (VCAM-1). Proliferation, damage, and apoptosis of HCAECs were examined using 3-(4,5-dimethylthiazol-2-yl)-2,5-diphenyl tetrazolium bromide, lactate dehydrogenase (LDH), and flow cytometry, respectively. mCRP suppressed VEGF and COX-2 expression and enhanced ICAM-2 and VCAM-1 expression in HCAECs, in both dose-dependent and time-dependent manner. Except at 100 µg/ml concentration and 20-hour or 24-hour incubation, pCRP had no apparent effects. mCRP but not pCRP induced HCAEC injury and phosphorylation of p38 MAPK, and the inhibitor SB203580 reversed the effects of mCRP. mCRP promotes injury and apoptosis of HCAECs through a p38 MAPK-dependent mechanism, which provides a new therapy for the injury of HCAECs in atherosclerosis.

## INTRODUCTION

At present cardiovascular disease has become the most common cause of death worldwide. The main contributing factor of cardiac death is atherosclerosis, which is defined as a chronic inflammatory disease in the arterial wall [1]. Atherosclerosis is exacerbated by certain cardiovascular risk factors, such as inflammatory response, elevated basal levels of cytokines, hypertension, diabetes, and obesity, of which inflammation plays an important role in plaque development and precipitation of clinical symptoms [2,3]. Atherosclerosis in its early stages is usually asymptomatic due to plaques forming over a prolonged period of time [4]. The atherosclerosis event is as an initial manifestation of the disease, including acute coronary diseases, myocardial infarction, and stroke [5-7].

C-reactive protein (CRP) belongs to the highly conserved family of calcium-dependent ligand-binding plasma proteins [8]. Pentameric CRP (pCRP), a disc of five identical subunits noncovalently bounded around a central pore, is present in the circulation [9]. It dissociates in a non-reversible manner into its non-soluble monomers to generate a potential active form [10]. CRP in atherosclerosis patients not only serves as a biomarker of cardiovascular disease risk but it also plays a part in mediating atherosclerosis by promoting arterial endothelial activation and mediating inflammatory reactions and the innate immune response [11,12]. Of note, monomeric CRP (mCRP) is considered to be involved in the innate immune system by mediating the complement cascade in angiogenesis and thrombosis [13]. In addition, the serum of patients with cardiovascular events has an increased level of CRP. Previous research had revealed that the CRP protein is expressed in macrophages and vascular smooth muscle cells in atheromatous plaques [14,15] and contributes to the progression and vulnerability of atherosclerotic lesions [16]. Therefore, CRP inhibition could represent a viable new approach to prevent and treat of cardiovascular diseases. Accordingly, CRP promotes endothelial dysfunction by inhibiting nitric oxide production to impair endothelial-dependent vascular relaxation [17,18]. A growing body of evidence suggests that CRP could promote endothelial dysfunction by inducing the release of monocyte-chemoattractant protein-1 [19]. However, the mechanism linking CRP and the development of atherosclerosis is poorly understood.

Research shows that p38 mitogen-activated protein kinase (p38MAPK) signaling pathway plays an important role in various inflammatory ocular diseases [20]. Activation of P38 MAPK/nuclear factor-kappa B (NF-κB) induces the production of key inflammatory mediators, such as tumor necrosis factor-alpha, interleukin (IL)-1β, and IL-6 [21]. Recent studies have also shown that MAPK-NF-κB pathway is an important component of cellular signal transduction in the development of atherosclerosis by regulating inflammatory gene expression [22]. Therefore, it is believed that p38MAPK is an obvious therapeutic target for chronic inflammatory diseases.

The present study was aimed at investigating the effects of CRP at the different concentrations and times on the expression of vascular endothelial growth factor (VEGF), cyclooxygenase-2 (COX-2), intercellular adhesion molecule-2 (ICAM-2), and vascular cell adhesion molecule-1 (VCAM-1), known as atherosclerosis-related genes in human coronary artery endothelial cells (HCAECs). Furthermore, we found that mCRP mediates p38MAPK-dependent expression of VEGF, COX-2, ICAM-2, and VCAM-1 protein in HCAECs, and this affects HCAECs injury. These findings reveal that p38 MAPK is a new mechanism for CRP-mediated HCAECs dysfunction, which may be a potential target for vascular disease.

## MATERIALS AND METHODS

### Cells treatment and transfection

HCAECs were purchased from Shanghai Zishi Biotechnology Co., Ltd. (Shanghai, China). Cells were cultured in endothelium growth medium (EGM-MV) (Clonetics Corporation, San Diego, CA, USA) supplemented with 10% FBS. Cells were treated with pCRP or mCRP in different concentrations and the time. The treatment was as followed: HCAECs in 12-well, 24-well or 96-well plates (confluence 97%, 28,000 cells/cm^2^) were incubated with pCRP or mCRP (0, 5, 10, 20, 50, and 100 μg/ml) for 4 hours, or cells were treated with pCRP or mCRP (10 μg/ml) for 0, 4, 8, 12, 16, 20, and 24 hours, and 0 μg/m and 0 hour as respective comparison group. In some experiments, HCAECs were prestimulated with the p38 MAPK inhibitor SB203580 (0.1 mol/L) (Calbiochem, La Jolla, CA) for 20 minutes before addition of pCRP or mCRP. At the indicated times, cells were collected and were processed according to the different experimental purpose. High-purity (99%) human pCRP was purchased from BindingSite (Birmingham, UK).

mCRP was obtained by dissociation of pCRP in 8 M urea-EDTA [23]; small interfering RNA (siRNA) for p38MAPK was purchased from RiboBio Inc. (Guangdong, China), and p38 MAPK siRNA and control-siRNA were transfected into HCAECs using Lipofectamine^™^ 2000 (Invitrogen, Carlsbad, CA, USA) according to the manufacturer’s protocol. This study was approved by the Ethics Committee of the First Affiliated Hospital of Anhui Medical University.

### Western blot analysis

Total protein (20 μg) was extracted from SB203580-pretreated or p38 MAPK siRNA-transfected or/and CRP-treated HCASCs. Proteins were separated in 12% SDS-PAGE, then transferred onto polyvinylidene fluoride membranes (Millipore, Billerica, USA). After membranes were blocked with 5% nonfat dry milk in Tris-buffered saline (pH 7.4) containing 0.02% Tween 20 (TBST) for 1.5 hours at room temperature, then incubated with antibodies against p38MAPK, p- p38MAPK, ICAM-2, VCAM-1, COX-2 (1:1,000), (all from Cell Signaling Technology); VEGF (1:200; Acris Antibodies, Herford, Germany); and GAPDH (1:5000, Abcam, USA) at 4°C overnight. The membranes were washed and incubated using horseradish peroxidase-conjugated immunoglobulin G antibody (Cell Signaling Technology) at 37°C for 2 hours. The bands were visualized using the Enhanced Chemiluminescence-Plus Western Blotting Detection System (Amersham, Cambridge, UK). The relative levels of the target proteins expression were normalized against that of the GAPDH.

### Apoptosis assays

HCAECs (2 × 10^6^ cells/6-cm dish) were treated as described above. Cells were then washed with phosphate buffer solution. The cells were incubated with an apoptosis assays kit (BD Pharmingen, San Diego, CA) in accordance with the manufacturer’s instructions. In brief, Annexin V-FITC (5 μL) and PI (5 μL) were mixed with the cells for 15 minutes at room temperature without light. Data were analyzed by flow cytometry (Beckman Coulter).

### 3-(4,5-dimethylthiazol-2-yl)-2,5-diphenyl tetrazolium bromide (MTT) assay

Cell proliferation was detected using MTT assay kit (Sigma, USA) according to the manufacturer’s instructions. Briefly, cells were cultured in 96-well plates (with 100 μl/well medium) in the absence or in the presence of SB203580 or p38 MAPK siRNA or/and CRP (10 μg/ml) for 24 hours. Then cells were incubated with 10 μl MTT (5 mg/ml) for 4 hours at 37°C. Formazan product was solubilized by the addition of 200 μl of dimethyl sulfoxide in dark to dissolve the formazan crystals for 30 minutes. Succinodehydrogenase activity was expressed as absorbance at a test wavelength of 570 nm using a microplate reader (Spectra Max M5, Molecular Device, USA).

### Lactate dehydrogenase (LDH) assay

Cell injury assay was performed using LDH (Sigma, USA); cells cultured in 96-well plates (with 100 μl/well medium) were treated as described above. LDH (50 μl) was added to cell supernatant from light for 30 minutes. LDH activity was expressed as absorbance at a test wavelength of 490 nm using a microplate reader (Spectra Max M5, Molecular Device, USA).

### Statistical analysis

Statistical analyses were performed by SPSS 19.0 software (IBM, Armonk, NY, USA) Results are expressed as mean ± SEM. Statistical comparisons were made by using Student’s *t-*test and ANOVA followed by the Dunnett’s or Tukey’s test. Values of *p* < 0.05 were considered significant for all tests. All experiments were done in triplicate and repeated at least thrice.

## RESULTS

### Effect of CRP on expression of VEGF, COX-2, ICAM-2, and VCAM-1 in HCAECs

**FIGURE S1:**
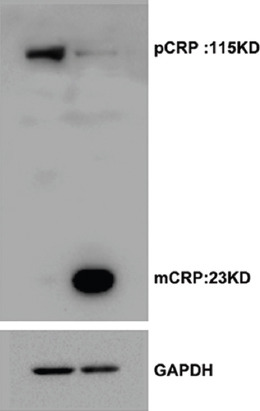
Purity identification for mCRP. Pentameric C-reactive protein (pCRP) and the products obtained from pCRP were tested by Western bolt.

**FIGURE 1:**
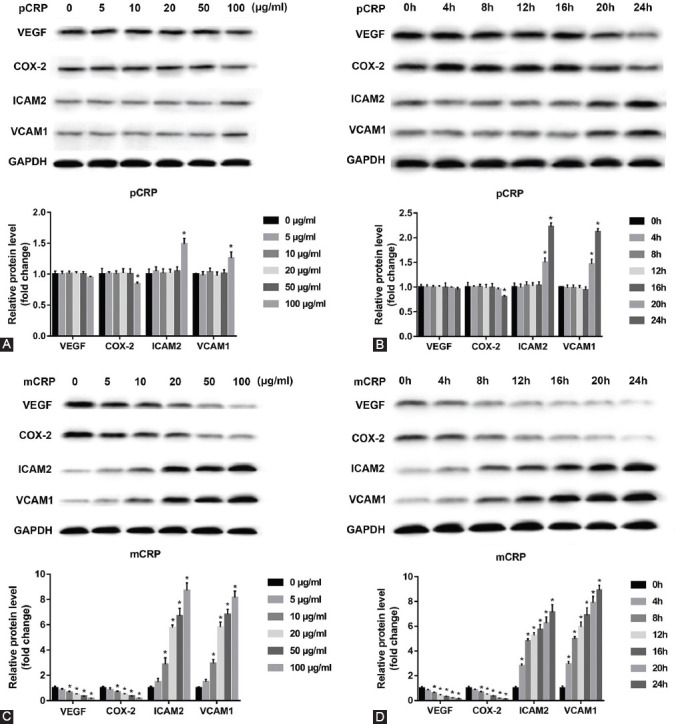
Effect of C-reactive protein (CRP) on levels of vascular endothelial growth factor (VEGF), cyclooxygenase-2 (COX-2), intercellular adhesion molecule-2 (ICAM-2), and vascular cell adhesion molecule 1 (VCAM-1) protein in human coronary artery endothelial cells (HCAECs). Representative pictures of Western blot of VEGF, COX-2, ICAM-2, and VCAM-1. (A) Serum-starved HCAECs were treated with pentameric CRP (pCRP) (5, 10, 20, 50, and 100 μg/ml) or vehicle for 4 hours. (B) Serum-starved HCAECs were treated with pCRP (10 μg/ml) or vehicle for 0, 4, 8, 12, 16, 20, and 24 hours. (C) Serum-starved HCAECs were treated with monomeric CRP (mCRP) (5, 10, 20, 50, and 100 μg/ml) or vehicle for 4 hours. (D) Serum-starved HCAECs were treated with mCRP (10 μg/ml) or vehicle for 0, 4, 8, 12, 16, 20, and 24 hours. *p < 0.05 versus control. Statistical comparisons were performed using ANOVA followed by the Dunnett’s test. n = 3–5 per group.

First, mCRP was produced by dissociation of pCRP. The result of Western blot showed the purity of the CRPs in the monomeric and pentameric form (Figure S1). Then, HCAECs treated with pCRP (Figure 1A) and mCRP (Figure 1C) (0, 5, 10, 20, 50, and 100 μg/ml) for 4 hours or with pCRP (Figure 1B) and mCRP (Figure 1D) (10 μg/ml) for different time points (0, 4, 8, 12, 16, 20, and 24 hours). As compared to 0 μg/ml or 0 hour, pCRP (100 μg/ml for 4 hours or 10 μg/ml for 20 or 24 hours) significantly reduced the level of COX-2, but increased ICAM-2 and VCAM-1 expression as demonstrated by Western blot (^*^p < 0.05, Figure 1A and B). However, pCRP had no significant effects on the protein levels of VEGF. In addition, we found that the level of VEGF and COX-2 protein in HCAECs was significantly decreased, but ICAM-2 and VCAM-1 were observably increased by mCRP treatment in both dose-dependent manner and time-dependent manner (^*^p < 0.05, Figure 1C and D).

### mCRP promotes HCAECs apoptosis

**FIGURE 2:**
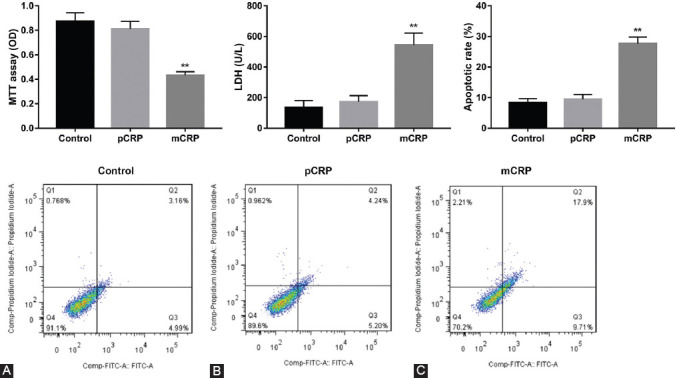
Monomeric C-reactive protein (mCRP) induced human coronary artery endothelial cells (HCAECs) damage. (A) 3-(4,5-dimethylthiazol-2-yl)-2,5-diphenyl tetrazolium bromide assay of HCAECs treated using pentameric C-reactive protein (pCRP) and mCRP. (B) Lactate dehydrogenase assay of HCAECs treated using pCRP and mCRP. (C) Representative immunoblot and quantification of analysis of HCAECs apoptosis by flow cytometry. **p < 0.01 versus controls. Statistical comparisons were made using ANOVA followed by the Dunnett’s test. n = 3–5 per group.

To investigate the functional significance of alteration of VEGF, COX-2, ICAM-2, and VCAM-1 protein, we detected the effect of CRP (10 μg/ml, 24 hours) on cell proliferation and apoptosis. mCRP induced a dramatical decrease in cell proliferation (Figure 2A, **p < 0.01 vs. controls), increase in cell injury(Figure 2B, **p < 0.01 vs. controls) and apoptosis(Figure 2C, **p < 0.01 vs. controls) as compared to controls, whereas in pCRP-pretreated HCAECs had no significant effects on the cell function. These data indicate that mCRP may be involved in the HCAEC injury.

### mCRP mediates p38 MAPK-dependent expression of VEGF, COX-2, ICAM-2, and VCAM-1 protein in HCAECs

**FIGURE 3:**
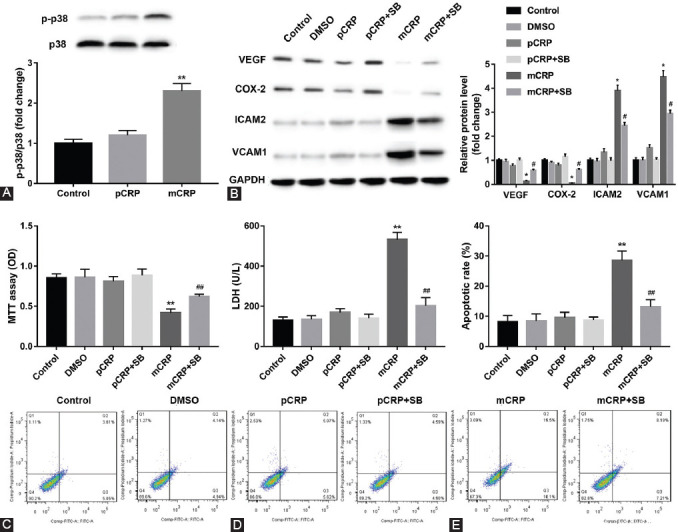
Effect of p38 mitogen-activated protein kinase (p38 MAPK) inhibition on monomeric C-reactive protein (mCRP) -mediated atherosclerosis-related molecular release. (A) Phosphorylation of p38 MAPK inducted by pentameric C-reactive protein (pCRP) and mCRP. *p < 0.05 versus control. Statistical comparisons were made using Dunnett’s test. (B) Human coronary artery endothelial cells (HCAECs) were prestimulated with SB203580 (p38 MAPK inhibitor) for 20 minutes and then were cultured with mCRP or pCRP (10 μg/ml) for 24 hours. *p < 0.05 versus control; ^#^*p* < 0.05 versus mCRP treatment group. Statistical comparisons were made using ANOVA followed by Tukey’s test. p38 MAPK inhibition on mCRP or pCRP-induced HCAECs damage. (C) 3-(4,5-dimethylthiazol-2-yl)-2,5-diphenyl tetrazolium bromide assay of HCAECs. (D) Lactate dehydrogenase assay of HCAECs. (E) The analysis of flow cytometry. *p < 0.05, **p < 0.01 versus control; ^#^*p* < 0.05, ^##^*p* < 0.01 versus mCRP treatment group. Statistical comparisons were made using ANOVA followed by Tukey’s test. n = 3–5 per group.

Exogenous pCRP and mCRP induced phosphorylation of p38 MAPK relative to untreate controls. As shown in Figure 3A, phosphorylation was markedly elevated in mCRP treatment group than control (**p < 0.01 vs. control). Furthermore, pretreatment of HCAECs with SB203580 increased mCRP-inhibited VEGF and COX-2 release at 24 hours, whereas decreased mCRP-induced ICAM-2 and VCAM-1 expression (Figure 3B, *p < 0.05 vs. control; ^#^*p* < 0.05 vs. mCRP treatment group). In addition, MTT showed that cell proliferation was significantly decreased. Conversely, cells damage and apoptosis were markedly increased in the mCRP treatment group than that in the control, as demonstrated by LDH and flow cytometry assay. However, inhibitor of p38 MAPK reversed these results (Figure 3C-E, **p < 0.01 vs. control; ^##^*p* < 0.01 vs. mCRP treatment group). It is noticeable that pCRP had no effect on p38 MAPK-regulated cell function.

### mCRP-induced HCAECs injury is mediated through triggering activation of p38 MAPK

**FIGURE 4:**
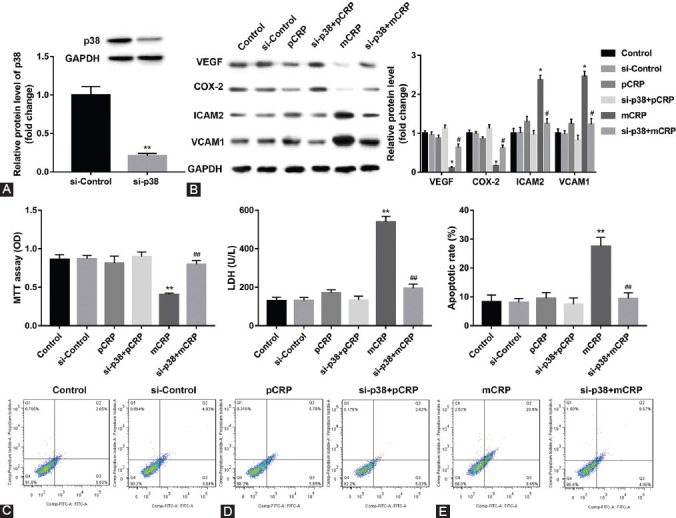
Monomeric C-reactive protein (mCRP) -induced cell injury and apoptosis mediated by p38 mitogen-activated protein kinase (p38MAPK) activation. (A) p38 MAPK siRNA inhibited mCRP or pentameric C-reactive protein (pCRP) -induced p38MAPK expression in human coronary artery endothelial cells (HCAECs). **p < 0.01 versus si-control. Statistical comparisons were made using Student’s *t*-test. (B) p38MAPK siRNA inhibited mCRP-induced CAM2 and vascular cell adhesion molecule 1 release and promoted mCRP-suppressed vascular endothelial growth factor and cyclooxygenase-2 release in HCAECs. (C) p38MAPK siRNA promoted mCRP-inhibited cell proliferation. (D-E) p38MAPK siRNA inhibited mCRP-induced cell injury and apoptosis. *p < 0.05, **p < 0.01 versus control; ^#^*p* < 0.05, ^##^*p* < 0.01 versus mCRP treatment group. Statistical comparisons were made using ANOVA followed by Tukey’s test. n = 3–5 per group.

Recent studies have demonstrated that CRP activates MAPKs (ERK1/2, JNK, and p38MAPK) in RAW264.7 cells. We detected whether p38MAPK interference accompanied the alteration of mCRP-induced HCAECs injury. HCAECs were first transfected using siRNA of p38MAPK. We found that HCAECs transfected with p38MAPK siRNA showed marked suppression of p38MAPK expression (Figure 4A, **p < 0.01 vs. si-control). Furthermore, inhibition of p38MAPK reversed mCRP-suppressed expression of VEGF and COX-2 and mCRP-induced ICAM-2 and VCAM-1 expression, as demonstrated by Western blot analysis, effects that were not exhibited in HCAECs transfected with control siRNA (Figure 4B, *p < 0.05 vs. control; ^#^*p* < 0.05 vs. si-control). Besides, the p38MAPK silencing ameliorated mCRP-induced cell injury (Figure 4C-E, **p < 0.01 vs. control; ^##^*p* < 0.01 vs. si-control).

## DISCUSSION

CRP has been implicated in predicting the activity and vulnerability of atheromatous plaque rupture [24]. It has been demonstrated that CRP is localized in atherosclerotic lesions to modulate the pathogenesis of atherosclerosis [16]. The CRP as an active mediator of atherosclerosis could promote arterial endothelial activation and macrophage recruitment [25]. The previous study has also shown that CRP has a higher expression level in atherosclerosis patients [24,26]. In addition, an intensive expression of CRP in macrophages and VSMCs was associated with stabilization of atherosclerotic plaque [27,28]. In our study, we found that VEGF and COX-2 protein expression was decreased and the expression of ICAM-2 and VCAM-1 was increased in HCAECs treated with mCRP, in both dose-dependent manner and time-dependent manner. Besides, mCRP induced a significant decrease in cell proliferation, increase in cell injury and apoptosis than that in the control group. However, the effect of pCRP-pretreated HCAECs had no significant effects on the atherosclerosis-related factors and cell damage. These data suggest that pCRP and mCRP exhibit different functions, and the CRP activity is expressed only when the circulatory pentameric structure of CRP is lost to form mCRP in disease states.

The pCRP dissociation into a monomer is one likely reason why pCRP regulates expression of atherosclerosis-related factors with the prolonging of time. In our research, the dramatic delay in pCRP effect on HCAECs confirms this assumption. Some of the pCRP was converted to mCRP on the HCAECs surface. Thus, it is confirmed that mCRP rather than pCRP participates in the progression of atherosclerotic lesions and the HCAEC injury. The previous study has indicated that CRP activates MAPKs through the Fcγ receptor [29]. Moreover, mCRP plays a pivotal role in chronic inflammatory diseases [30,31]. mCRP could activate the phosphorylation of p38 MAPK in RAW264.7 cells [30]. We have recently demonstrated that exogenous mCRP significantly induced phosphorylation of p38 MAPK. Furthermore, p38 MAPK inhibition increased mCRP-restrained VEGF and COX-2 release and it decreased mCRP-induced ICAM-2 and VCAM-1 expression. In addition, MTT, LDH, and flow cytometry assay showed that mCRP treatment suppressed cell proliferation, induced cell damage and apoptosis, but inhibitor of p38 MAPK reverses these results. These results indicate that mCRP mediates p38 MAPK-dependent expression of atherosclerosis-related protein in HCAECs.

Our results further indicate that intervention of p38MAPK ameliorated mCRP-induced cell injury, and mCRP-induced HCAECs injury is mediated by activating of p38 MAPK.

## CONCLUSION

our results indicate that mCRP is involved in the injury of HCAECs, but pCRP does not have such effect in HCAECs. The mCRP effects on HCAECs are mediated by activation of the p38 MAPK pathway. mCRP may be a potential regulator of signaling pathways associated with ICAM-2, VCAM-1, VEGF, and COX-2. These findings indicate that mCRP rather than pCRP may contribute to the development of endothelium cell injury in atherosclerosis.
